# Elevated Preoperative Serum CA125 Predicts Larger Tumor Diameter in Patients with Hepatocellular Carcinoma and Low AFP Levels

**DOI:** 10.1155/2019/6959637

**Published:** 2019-09-29

**Authors:** Sanshun Zhou, Zusen Wang, Manjiang Li, Liqun Wu

**Affiliations:** ^1^Liver Disease Center, The Affiliated Hospital of Qingdao University, Qingdao, Shandong, China; ^2^Department of Hepatobiliary and Pancreatic Surgery Center, The Affiliated Hospital of Qingdao University, Qingdao, Shandong, China

## Abstract

**Aim:**

Little is known about the association between cancer antigen 125 (MUC16/CA125) concentrations and tumor diameter of patients with hepatocellular carcinoma (HCC) and low AFP levels. To fill this gap in our knowledge, we conducted a retrospective study of 427 patients with HCC with AFP ≤200 ng/mL who underwent R0 resection at our center.

**Methods:**

The associations between CA125 concentrations and patients' clinicopathological characteristics were analyzed. Survival vs CA125 levels was also evaluated between patient groups with CA125 ≤30 U/mL or CA125 >30 U/mL. Independent risk factors of disease-free survival (DFS) and overall survival (OS) were analyzed with Cox hazard regression model.

**Results:**

Elevated preoperative serum CA125 was significantly associated with maximal tumor diameter (MTD) >5 cm and female sex (*P* < 0.001 and *P*=0.044, respectively). The DFS and OS of patients with CA125 ≤30 U/mL (*n* = 392) were significantly higher compared with those with CA125 >30 U/mL (*n* = 35) (*P*=0.003 and *P*=0.001 respectively). Multivariate analysis revealed that MTD >5 cm was an independent risk factor of DFS (HR = 1.891, 95% CI: 1.379–2.592, *P* < 0.001) and OS (2.709, 1.848–3.972, *P* < 0.001).

**Conclusions:**

In conclusion, elevated preoperative serum CA125 predicted larger tumor diameter and poor prognosis after patients with HCC with AFP ≤200 ng/mL underwent R0 resection, which may be explained by the elevation of the preoperative serum CA125 level significantly associated with MTD>5 cm.

## 1. Introduction

Liver cancer is the sixth most commonly diagnosed cancer and the fourth leading cause of cancer death worldwide in 2018 [1]. Hepatocellular carcinoma (HCC) is one of the most frequently occurring types of liver cancer, with more than 700,000 new cases per year worldwide [2]. It is well known that measurement of tumor size in patients with HCC before hepatectomy mainly depends on imaging examination. However, it is often affected by the physiological activities of some organs, such as respiratory movement, cardiac beat, digestive tract peristalsis, and so on, forming artifacts and affecting observation. Ultrasound (US) is the first line method for detection and characterization of abdominal pathologies [3], but abdominal organ assessment with US is specifically associated with significant limitations in image quality and incomplete visualization of organs. Computed tomography (CT) and magnetic resonance (MR) imaging have been researched for the detection and characterization of hepatocarcinogenesis [4]. Particularly when using contrast agents, they can provide vivid liver parenchyma images. In spite of this, the necessity of repetition and radiation may be burdensome to patients.

Recent advances in diagnostic techniques and improved surgical methods have improved early diagnosis and the resection rates of HCC. However, the long-term prognosis of patients with HCC remains poor, and the recurrence rate at 5 years is as high as 70% [5]. Unfortunately, this dismal situation can be attributed, in part, to the lack of an effective indicator for evaluating the prognosis of these patients. We reasoned that AFP might serve this purpose because it is a relatively specific marker for HCC and is highly valuable for diagnosis and prognosis. However, certain patients with HCC have normal or insignificant increases in AFP levels in the early and advanced stages of disease [6, 7]. The AFP level is also affected by age [8], viral hepatitis or liver fibrosis [9], and some neurodegenerative diseases [10]. Therefore, effective tools are required to predict tumor diameter and prognosis of HCC patients with preoperative serum AFP ≤200 ng/mL [11].

Moreover, circulating CA125 (MUC16) is often used as a marker to diagnose ovarian tumors [12] and a prognostic marker for pancreatic cancer [13] and for the presence of cirrhosis [14]. The utility of CA125 in the diagnosis of HCC has been explored [15]. However, there are few reports of the relationship between CA125 and tumor size or prognosis in patients with HCC. To fill this gap in our knowledge, here we aimed to evaluate the association between preoperative serum CA125 level and tumor diameter, also prognostic significance of CA125 for HCC.

## 2. Methods

We retrospectively analyzed the clinicopathological data of 427 patients with HCC with AFP ≤200 ng/mL (366 men, 61 women; median age, 57.0 years) who underwent R0 resection at the Affiliated Hospital of Qingdao University from January 2009 to December 2015. Out of a total of 427 patients, 359 were HBV-related HCC patients. They were all treated with nucleic acid analog for hepatitis B. All patients signed informed consent forms before surgery. The Affiliated Hospital of Qingdao University Ethics Committee approved this study, which complies with the 2008 Declaration of Helsinki of the World Medical Association.

Inclusion criteria were as follows: (a) tumor markers CA125 and AFP measured simultaneously before surgery and AFP ≤200 ng/mL; (b) patients underwent R0 resection, HCC confirmed by pathology; (c) no history of preoperative anticancer treatment including TACE, percutaneous radiofrequency ablation (PRFA), or percutaneous ethanol injection (PEI); (d) complete clinicopathological and follow-up data. Exclusion criteria were as follows: (a) preoperative history of other malignant neoplasms or the presence of ascites; (b) recent infection or abdominal surgery; (c) one of the two tumor markers not measured before surgery; (d) postoperative pathology confirmed hepatobiliary cell carcinoma or mixed cell carcinoma; (e) missing clinicopathological data.

Blood samples were obtained 1 to 7 days before surgery. Serum was obtained for analyses of CA125 and AFP. The concentration of serum CA125 was detected using an ELISA method, with 30 U/mL as the cutoff value. AFP was also detected using an ELISA method, with 200 ng/mL as the cutoff value. Patients were divided into two groups: patients with preoperative serum CA125 concentrations ≤30 U/mL (*n* = 392) and patients with preoperative serum CA125 level >30 U/mL (*n* = 35).

### 2.1. Follow-Up

Patients underwent regular follow-up examinations after surgery. Medical assessment was performed once a month within the first 3 months, and once every 3 months thereafter. Evaluations included serum AFP; liver function; digestive system ultrasound; and enhanced computed tomography, enhanced magnetic resonance imaging, or both, when recurrence was suspected. Follow-up ended on December 31, 2017 or upon the patient's death.

### 2.2. Statistical Analysis

All statistical analyses were performed using SPSS 19.0 software (SPSS Inc., Chicago, IL, USA). Normally distributed quantitative variables are presented as the mean ± standard deviation. For nonnormally distributed variables, we used median and interquartile range (IQR). Categorical data are summarized using ratios and percentages. Differences between proportions of categorical data were compared using Pearson's chi-square test or Fisher's exact test where appropriate. Disease-free survival and overall survival were assessed using Kaplan–Meier analysis, and log-rank tests were used to evaluate differences between groups. Multivariate analyses of survival were performed using the Cox proportional hazards model. *P* < 0.05 was considered statistically significant.

## 3. Results

The candidate study population included 596 consecutive patients with HCC who underwent R0 resection with curative intent. We excluded 169 (28%) patients who did not meet the inclusion criteria. Included patients' (*n* = 427) baseline demographics as well as their clinical and pathological characteristics are shown in Table 1.

We analyzed the correlation between CA125 concentrations and clinicopathological characteristics of the CA125 ≤30 U/mL and the CA125 >30 U/mL groups. We found that elevated preoperative serum CA125 concentrations were associated with maximal tumor diameter (MTD) >5 cm and female sex (Table 2). With the exceptions of sex and MTD, other variables did not significantly differ between patients with CA125 ≤30 U/mL (*n* = 392) and CA125 >30 U/mL (*n* = 35).

**Figure 1:**
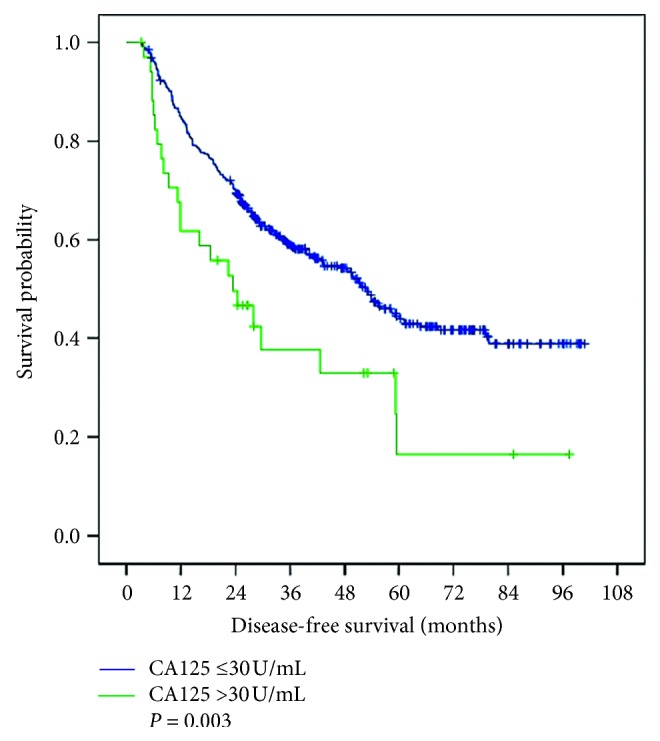
Disease-free survival of patients with CA125 ≤30 U/mL and CA125 >30 U/mL.

**Figure 2:**
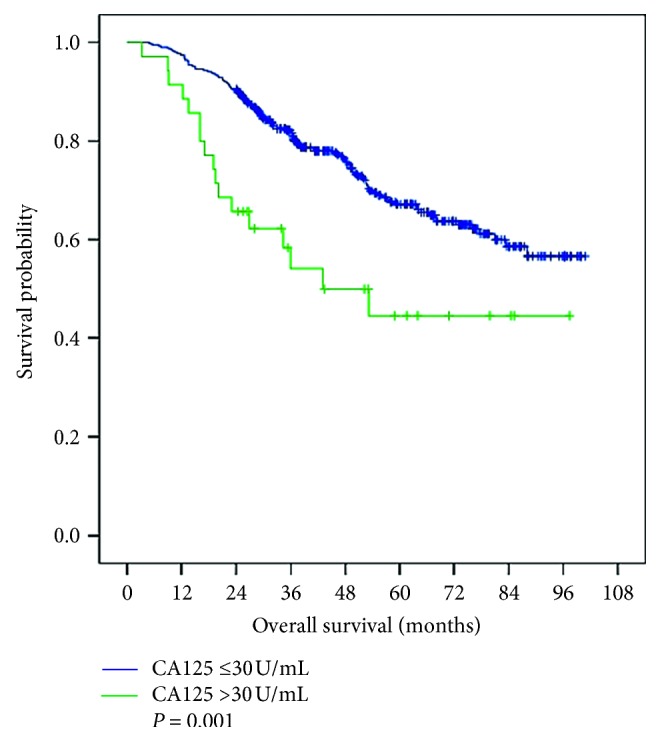
Overall survival of patients with CA125 ≤30 U/mL and CA125 >30 U/mL.

The median follow-up time was 46.2 months (range, 3.3–100.8 months). There were 135 deaths during follow-up. Kaplan–Meier analyses revealed that the 1-, 2-, and 5-year disease-free survival (DFS) rates among patients with CA125 ≤30 U/mL were 84.9%, 69.9%, and 43.9%, respectively. Among those with CA125 >30 U/mL, these values were 61.8%, 49.7%, and 16.5%, respectively. The median disease-free survival times were 53.2 months and 23.4 months, respectively (*P*=0.003, Figure 1). Moreover, 1-, 2-, and 5-year overall survival (OS) rates among patients with CA125 ≤30 U/mL were 97.4%, 90.6%, and 67.2%, respectively. These values were significantly better compared with those among patients with CA125 >30 U/mL (91.4%, 65.7%, and 44.5%, respectively). The median overall survival times of the CA125 ≤30 U/mL and the CA125 >30 U/mL groups were 96.0 months and 53.3 months, respectively (*P*=0.001, Figure 2).

Tables 3 and 4 show the results of univariate and multivariate Cox regression analyses of DFS and OS. Multivariate Cox regression analysis demonstrated that Cirrhosis (HR = 1.708, 95% CI: 1.079–2.703, *P*=0.022), Child-Pugh B (6.373, 3.289–12.349, *P* < 0.001), MTD >5 cm (1.891, 1.379–2.592, *P* < 0.001), TNM stages (1.358, 1.052–1.753, *P*=0.019) were independent risk factors for DFS. Moreover, Cirrhosis (HR = 2.615, 95% CI: 1.266–5.407, *P*=0.009), Child-Pugh B (4.942, 2.395–10.198, *P* < 0.001), MTD >5 cm (2.709, 1.848–3.974, *P* < 0.001), and TNM stages (1.471, 1.109–1.951, *P*=0.007) were also found to be independent risk factors in Cox regression for OS.

## 4. Discussion

Here we analyzed the association between tumor size, survival of patients with HCC who underwent R0 resection, and preoperative serum CA125 levels. We found that elevated preoperative serum CA125 concentrations were associated with larger tumor diameter and poor prognosis among patients, although there was no significant increase in preoperative serum AFP concentrations. Our study therefore provides a new approach for predicting tumor size and the prognosis of HCC patients whose preoperative serum AFP concentrations are not significantly elevated.

CA125 was first identified in a screen of monoclonal antibodies raised against the ovarian cancer cell line OVCA433 [16, 17]. Therefore, CA125 is the most commonly used tumor marker for the detection of epithelial ovarian cancer. Numerous studies show that CA125 can provide a reference value for prognostic evaluation of patients with various tumors. Analysis of preoperative serum CA125 levels and postoperative survival of 90 patients with epithelial ovarian cancer suggests that the preoperative CA125 concentration serves as a prognostic factor for overall survival [18]. Further, high preoperative concentrations of serum CA19-9, CA125, or both may predict an increased risk of recurrent disease in patients with resectable lung adenocarcinoma [19]. Moreover, CA125 serves as a prognostic marker for patients with pancreatic cancer, and mechanistic studies found that the KRAS/MYC axis drives the upregulation of MUC16/CA125 [13].

To our knowledge, the relationship between preoperative serum CA125 concentrations and tumor size and the prognosis of patients with HCC has not been studied. The present study found that high preoperative serum CA125 concentrations were significantly associated with MTD >5 cm and female sex (Table 2). Consistent with our data are findings that serum CA125 concentrations are significantly higher in patients with endometrial cancer with larger tumor diameters [20]. We carefully excluded patients with other possible causes of elevated preoperative serum CA125, including those with preoperative complications caused by other malignant tumors, recent infections, the presence of ascites, or abdominal surgery. These conditions can increase tumor marker levels, particularly those of CA125, which may act as confounding factors, because of the detection of serum CA125 level having advantages of lower cost, shorter period, and better repeatability. We suggest that CA125 could be used as an alternative marker for detecting tumor size in a patient with HCC before hepatectomy. Study of patients with ovarian cancer found that CA125 inhibits the anticancer immune response [21]. We suspect that the mechanism underlying the association between high CA125 levels and MTD >5 cm may be the same. Serum CA125 concentrations tend to increase in patients with ovarian cancer. In the entire patients, 35 patients had elevated concentrations of CA125, nine were female, and none had a history of ovarian cancer. We reasoned therefore that the rise of CA125 concentrations observed here was related to female sex, not ovarian cancer.

Our study present also shows that elevated preoperative serum CA125 concentrations (>30 U/mL) were associated with poor prognosis of patients, although there was no significant increase in preoperative AFP concentrations (≤200 ng/mL) (Figures 1 and 2). However, further studies are required to determine the mechanism. Tumor progression is not only determined by the intrinsic characteristics of the tumor but also by the local and systemic tumor environment [22], tumor-associated Tregs are important immune cells in the tumor microenvironment [23]. It has been reported that tumor-associated Treg promote tumor cell escape from the immune system [24], MUC16/CA125 can repress antitumor immune responses through inhibiting NK cell or T cell function in many cancers [25–27]. There may be a similar phenomenon in HCC.

A new study has shown that there is a serum increase in CA125 levels in patients with cirrhosis [14]. For the whole cohort, multivariate Cox regression analysis demonstrated that Cirrhosis, Child-Pugh B, TNM stages, and MTD >5 cm were independent risk factors for DFS and OS in the present study (Tables 3 and 4), while there were no differences in the prevalence of cirrhosis, Child-Pugh classification, and TNM stages between patient groups with CA125 ≤30 U/mL and CA125 >30 U/mL (Table 2). So, we excluded the possibilities that reduced survival in patients with elevated CA125 in the present study could be consequences of the three factors. As compared with the CA125 <30 U/mL group, MTD >5 cm were more proportions in the CA125 >30 U/mL group (63% vs 22%, *P* < 0.001). We suspect that the mechanism underlying the association between poor prognosis after patients with HCC with AFP ≤200 ng/mL underwent R0 resection and high CA125 levels may be this possibility. Despite a significant gender difference between the two groups (Table 2), gender is not a prognostic factor either by univariate or multivariate analyses of survival (Tables 3 and 4). Although serum CA125 is not an independent prognostic factor for patients with HCC, preoperative serum CA125 is more easily measured before hepatectomy and, when used in combination with pathology, imaging studies or other serum markers, may help in the assessment of prognosis after surgery.

The main limitation of the present study is its retrospective design. Second, only the effects of preoperative serum CA125 concentrations on tumor size and prognosis were analyzed, because there were many confounding factors that likely influence the CA125 concentrations after surgery, such as ascites [28] and insufficient liver function [29]. Thus, we did not investigate the association of postoperative CA125 concentrations with tumor size or prognosis. Third, the study included a relatively small number of patients, and our study population with elevated concentrations of CA125 was not so large. Fourth, this study showed that elevated preoperative CA125 was associated with MTD >5 cm in patients with HCC and low AFP levels, but there was no accurate quantitative evaluation of them.

## 5. Conclusion

In conclusion, elevated preoperative serum CA125 predicted larger tumor diameter in patients with HCC with AFP ≤200 ng/mL and may help in the assessment of prognosis after surgery. Patients with high preoperative CA125 levels should be carefully and reasonably managed.

## Figures and Tables

**Table 1:** Baseline characteristics of the entire patients (*n* = 427).

Characteristics	No. of patients	Proportion (%)
Age (years), mean ± SD	56.7 ± 9.5	
Gender		
Male	366	85.7
Female	61	14.3
Cirrhosis		
Yes	366	85.7
No	61	14.3
Virus hepatitis		
HBV	359	84.1
HCV	8	1.9
Non-HBC, non-HCV	60	14.0
Antiviral treatment^*∗*^		
Yes	359	84.1
No	68	15.9
Child-Pugh		
A	415	97.2
B	12	2.8
ALT (U/L), median (IQR)	36.0 (25.0–51.0)	
T-Bil (umol/L), median (IQR)	14.8 (11.2–19.4)	
Anatomical resection		
Yes	146	34.2
No	281	65.8
Surgical margin (mm)		
≤10	364	85.2
>10	63	14.8
MTD (cm)		
≤5	320	74.9
>5	107	25.1
Number of nodules		
1	363	85.0
>1	64	15.0
Microvascular invasion		
Yes	91	21.1
No	336	78.7
Differentiation		
Well	50	11.7
Mod, poor	377	88.3
TNM stage^*∗∗*^		
I	328	76.8
II	60	14.1
III	39	9.1

HBV, viral hepatitis B; HCV, viral hepatitis C; ALT, alanine transaminase; T-Bil, total bilirubin; MTD, maximal tumor diameter; IQR, interquartile range. ^*∗*^In this study, out of a total of 427 patients, 359 were HBV-related HCC patients. They were all treated with nucleic acid analog for hepatitis B. ^*∗∗*^TNM stages were assigned according to the criteria described in the AJCC Cancer Staging Manual (8th Edition).

**Table 2:** Comparison of clinicopathological characteristics between two groups stratified by CA125 level (*n* = 427).

Characteristics	CA125 ≤30 U/mL (*n* = 392)	CA125 >30 U/mL (*n* = 35)	*P*
Age (years) (≤65/>65)	325/67	31/4	0.389
Gender (male/female)	340/52	26/9	0.044
Cirrhosis (yes/No)	335/57	31/4	0.614
Virus hepatitis (HBV/HCV/non-HBV, non-HCV)	332/8/52	27/0/8	0.184
Child-Pugh (A/B)	383/9	32/3	0.074
ALT (U/L) (≤60/>60)	323/69	30/5	0.619
T-Bil (Umol/L) (≤20/>20)	304/88	26/9	0.659
Anatomical resection (yes/no)	130/262	16/19	0.134
Surgical margin (mm) (≤10/>10)	335/57	29/6	0.677
MTD (cm) (≤5/>5)	307/85	13/22	<0.001
Number of nodules (1/>1)	332/60	31/4	0.538
Microvascular invasion (yes/no)	82/310	9/26	0.507
Differentiation (well/mod, poor)	48/344	2/33	0.381
TNM stage^*∗*^			
I	304	24	0.115
II	56	4	
III	32	7	

HBV, viral hepatitis B; HCV, viral hepatitis C; ALT, alanine transaminase; T-Bil, total bilirubin; MTD, maximal tumor diameter; IQR, interquartile range. ^*∗*^TNM stages were assigned according to the criteria described in the AJCC Cancer Staging Manual (8th Edition).

**Table 3:** Univariate and multivariate analyses of DFS in the entire patients (*n* = 427).

Clinicopathological parameters	Univariate analysis		Multivariate analysis	
	HR (95% CI)	*P*	HR (95% CI)	*P*
Age (years) (≤65/>65)	1.078 (0.757–1.536)	0.676		
Gender (male/female)	0.685 (0.448–1.047)	0.080		
Cirrhosis (yes/no)	1.596 (1.017–2.506)	0.042	1.708 (1.079–2.703)	0.022
Virus hepatitis (HBV/HCV/non-HBV, non-HCV)	0.954 (0.834–1.091)	0.489		
Child-Pugh (A/B)	6.454 (3.366–12.374)	<0.001	6.373 (3.289–12.349)	<0.001
ALT (U/L) (≤60/>60)	1.237 (0.886–1.726)	0.212		
T-Bil (Umol/L) (≤20/>20)	0.973 (0.678–1.397)	0.884		
CA125 (U/mL) (≤30/>30)	1.913 (1.241–2.950)	0.003		
Anatomical resection (yes/no)	0.788 (0.591–1.050)	0.104		
Surgical margin (mm) (≤10/>10)	0.706 (0.472–1.056)	0.090		
MTD (cm) (≤5/>5)	2.085 (1.574–2.763)	<0.001	1.891 (1.379–2.592)	<0.001
Number of nodules (1/>1)	2.092 (1.509–2.899)	<0.001		
Microvascular invasion (yes/no)	1.536 (1.122–2.102)	0.007		
Differentiation (well/mod, poor)	1.175 (0.769–1.796)	0.455		
TNM stage^*∗*^(I/II/III)	1.808 (1.504–2.147)	<0.001	1.358 (1.052–1.753)	0.019

HBV, viral hepatitis B; HCV, viral hepatitis C; ALT, alanine transaminase; T-Bil, total bilirubin; MTD, maximal tumor diameter; IQR, interquartile range. ^*∗*^TNM stages were assigned according to the criteria described in the AJCC Cancer Staging Manual (8th Edition).

**Table 4:** Univariate and multivariate analyses of OS in the entire patients (*n* = 427).

Clinicopathological parameters	Univariate analysis		Multivariate analysis	
	HR (95% CI)	*P*	HR (95% CI)	*P*
Age (years) (≤65/>65)	1.034 (0.655–1.632)	0.887		
Gender (male/female)	0.865 (0.513–1.458)	0.586		
Cirrhosis (yes/no)	2.313 (1.313–4.732)	0.022	2.615 (1.266–5.401)	0.009
Virus hepatitis (HBV/HCV/non-HBV, non-HCV)	1.036 (0.877–1.224)	0.679		
Child-Pugh (A/B)	5.782 (3.013–11.099)	<0.001	4.942 (2.395–10.198)	<0.001
ALT (U/L) (≤60/>60)	1.562 (1.050–2.323)	0.028		
T-Bil (Umol/L) (≤20/>20)	0.687 (0.413–1.142)	0.147		
CA125 (U/mL) (≤30/>30)	2.266 (1.361–3.771)	0.002		
Anatomical resection (yes/no)	0.746 (0.513–1.086)	0.127		
Surgical margin (mm) (≤10/>10)	0.498 (0.275–0.901)	0.021		
MTD (cm) (≤5/>5)	3.013 (2.140–4.243)	<0.001	2.709 (1.848–3.972)	<0.001
Number of nodules (1/>1)	2.064 (1.381–3.087)	<0.001		
Microvascular invasion (yes/no)	1.583 (1.063–2.358)	0.024		
Differentiation (well/mod, poor)	1.357 (0.766–2.404)	0.296		
TNM stage^*∗*^ (I/II/III)	2.175 (1.745–2.712)	<0.001	1.471 (1.109–1.951)	0.007

HBV: viral hepatitis B; HCV, viral hepatitis C; ALT, alanine transaminase; T-Bil, total bilirubin; MTD, maximal tumor diameter; IQR, interquartile range. ^*∗*^TNM stages were assigned according to the criteria described in the AJCC Cancer Staging Manual (8th Edition).

## Data Availability

The data used to support the findings of this study are available from the corresponding author upon request.
